# Medical expenditure for lung cancer in China: a multicenter, hospital-based retrospective survey

**DOI:** 10.1186/s12962-021-00306-3

**Published:** 2021-08-17

**Authors:** Xin Zhang, Ju-Fang Shi, Guo-Xiang Liu, Jian-Song Ren, Lan-Wei Guo, Wei-Dong Huang, Lin-Mei Shi, Yi Ma, Hui-Yao Huang, Ya-Na Bai, Xian-Zhen Liao, A-Yan Mao, Xiao-Jie Sun, Xin-Yu Zhu, Qi Zhou, Ji-Yong Gong, Jin-Yi Zhou, Yu-Qin Liu, Ling Mai, Bing-Bing Song, Lin Zhu, Xiao-Jing Xing, Ling-Bin Du, Xiao Qi, Xiao-Hua Sun, Shou-Ling Wu, Ying Ren, Rong Cao, Li Lan, Pei-An Lou, Kai Zhang, Jie He, Min Dai

**Affiliations:** 1 School of Health Management, Harbin Medical University, 194 Xuefu Road, Nangang District, Harbin, 150081 China; 2 Office of Cancer Screening, National Cancer Center/National Clinical Research Center for Cancer/Cancer Hospital, Chinese Academy of Medical Sciences and Peking Union Medical College, 17 Panjiayuan South Lane, Chaoyang District, Beijing, 100021 China; 3 Department of Cancer Epidemiology, The Affiliated Cancer Hospital of Zhengzhou University, Henan Cancer Hospital, Zhengzhou, 450008 China; 4 Institute of Epidemiology and Health Statistics, Lanzhou University, Lanzhou, 730000 China; 5 Hunan Office for Cancer Control and Research, Hunan Provincial Cancer Hospital, Changsha, 410006 China; 6 Public Health Information Research Office, Institute of Medical Information, Chinese Academy of Medical Sciences, Beijing, 100020 China; 7 Center for Health Management and Policy, Key Lab of Health Economics and Policy, Shandong University, Jinan, 250012 China; 8 Chongqing Office for Cancer Control and Research, Chongqing Cancer Hospital, Chongqing, 400030 China; 9 Science and Education Department of Public Health Division, Shandong Tumor Hospital, Jinan, 250117 China; 10 Jiangsu Provincial Center for Disease Control and Prevention, Institute of Chronic Non-Communicable Diseases Prevention and Control, Nanjing, 210009 China; 11 Cancer Epidemiology Research Center, Gansu Provincial Cancer Hospital, Lanzhou, 730050 China; 12 Department of Institute of Tumor Research, The Affiliated Cancer Hospital of Zhengzhou University, Henan Cancer Hospital, Zhengzhou, 450008 China; 13 Heilongjiang Office for Cancer Control and Research, Affiliated Cancer Hospital of Harbin Medical University, Harbin, 150081 China; 14 Teaching and Research Department, Affiliated Cancer Hospital of Xinjiang Medical University, Urumqi, 830011 China; 15 Liaoning Office for Cancer Control and Research, Liaoning Cancer Hospital & Institute, Shenyang, 110042 China; 16 Zhejiang Office for Cancer Control and Research, Zhejiang Cancer Hospital, Hangzhou, 310022 China; 17 Department of Occupational Medicine, Tangshan People’s Hospital, Tangshan, 063001 China; 18 Ningbo Clinical Cancer Prevention Guidance Center, Ningbo NO.2 Hospital, Ningbo, 315010 China; 19 Health Department of Kailuan Group, Kailuan General Hospital, Tangshan, 063000 China; 20 Urban Office of Cancer Early Detection and Treatment, Tieling Central Hospital, Tieling, 112000 China; 21 Department of Health Policy and Economic Research, Guangdong Provincial Institute of Public Health, Guangzhou, 511430 China; 22 Institute of Chronic Disease Prevention and Control, Harbin Center for Disease Control and Prevention, Harbin, 150056 China; 23 Department of Control and Prevention of Chronic Non-Communicable Diseases, Xuzhou Center for Disease Control and Prevention, Xuzhou, 221006 China

**Keywords:** Lung cancer, Medical expenditure, Cost, China

## Abstract

**Background:**

Lung cancer is the most prevalent cancer, and the leading cause of cancer-related deaths in China. The aim of this study was to estimate the direct medical expenditure incurred for lung cancer care and analyze the trend therein for the period 2002–2011 using nationally representative data in China

**Methods:**

This study was based on 10-year, multicenter retrospective expenditure data collected from hospital records, covering 15,437 lung cancer patients from 13 provinces diagnosed during the period 2002–2011. All expenditure data were adjusted to 2011 to eliminate the effects of inflation using China’s annual consumer price index.

**Results:**

The direct medical expenditure for lung cancer care (in 2011) was 39,015 CNY (US$6,041) per case, with an annual growth rate of 7.55% from 2002 to 2011. Drug costs were the highest proportionally in the total medical expenditure (54.27%), followed by treatment expenditure (14.32%) and surgical expenditure (8.10%). Medical expenditures for the disease varied based on region, hospital level, type, and stage.

**Conclusion:**

The medical expenditure for lung cancer care is substantial in China. Drug costs and laboratory test are the main factors increasing medical costs.

## Introduction

Lung cancer has been the most common and deadly malignancy in the world for several decades [1]. In China, it is also the most prevalent cancer, estimated to be responsible for 20% of all new cancer cases in 2015. Furthermore, it is the leading cause of death from cancer (27.0%) [2]. The number of deaths from trachea, bronchus, and lung cancers rose from 260,200 in 1990 to 742,858 in 2018, in China [3, 4]. Disability-adjusted life-years (DALYs) of lung cancer were estimated at 15,284,700, accounting for 24.3% of cancer DALYs in 2017, in China [5]. Because of the high incidence and mortality rates, the costs associated with lung cancer treatment have created a heavy economic burden on health care resources [6, 7].

Accurate estimates of cancer costs are necessary for health care financing and cost-effectiveness analyses of relevant control interventions. However, medical costs associated with lung cancer treatment have not been conclusively demonstrated. Previous studies have contributed to estimations of such costs, but these findings have been limited to estimate the medical expenditure of single center, a certain type or therapy method because of the lack of reliable sampling and representative data in China [8–11]. A large public health service project, Cancer Screening Program in Urban China (CanSPUC), provided an opportunity to estimate the medical expenditure of cancer and their long-term trend. The CanSPUC was launched in August 2012 and supported financially by Chinese central government [12]. Lung cancer is one of the six targeted cancers of CanSPUC.

This aim of this study was to present nationwide representative estimates of hospital treatment expenditures associated with lung cancer care based a multicenter, hospital-based cross-sectional retrospective survey conducted by CanSPUC. This study provided a comprehensive breakdown of expenditures for different characteristics and explored the relationships between medical expenditures and geographic regions, hospital characteristics, patient characteristics, and therapy types. A 10-year trend analysis of the overall expenditure and its components was also conducted to present the change in the economic burden of lung cancer.

## Methods

### Data sources and sample size

As a part of CanSPUC program, a multicenter, hospital-based survey was conducted to estimate the medical expenditure incurred for cancer diagnoses and treatments in 13 provinces across China from 2012 to 2014. For each province, 1200 cases were sampled from 2002 to 2011 (120 cases each calendar year). The first case discharged on December 31 of the year was enrolled; then, based on the discharged date, 120 eligible cases were obtained for each hospital by continually moving back case by case. In order to ensure enough cases for subgroup analysis, the proportion of clinical stage (I–IV) and gender samples were required to keep balanced. A total of 15,437 cases of lung cancer patients were sampled from 37 hospitals in 13 cities in the eastern, central, and western regions—accounting for more than a third of China’s provinces (Table 1). Hospitals in China were divided into three tiers (primary, secondary, and tertiary) based on the level of service provision, medical technology, medical equipment, management, and medical quality. These three grades were further subdivided into three subsidiary levels: A, B, and C. This resulted in a total of nine levels, with tertiary A (3A) being the highest comprehensive level. In addition, hospitals were divided into general hospitals and specialized hospitals depending on the type of service provision. Clinical and medical expense information was extracted covering inpatient and outpatient visits of patients with lung cancer in the investigated hospitals.

**Table 1:** Summary of the survey sites and hospitals in 13 provinces in China

Province	General information at provincial level		Specific information on study sites and hospitals involved			
	Population size in 2011^a^, 10 000	GDP per capita in 2011^a^, CNY	City or cities involved	Total number of hospitals involved	No.( level) of general hospital involved	No./level of specialized hospital involved
Eastern region						
Guangdong	10,505	50,807	5 (Five cities ^b^)	6	6 (3A)	0
Shandong	9637	47,335	1 (Jinan)	1	0	1 (3A)
Beijing	2019	81,658	1 (Beijing)	5	3 (2 × 3A/1 × 3B or less)	2 (3A)
Zhejiang	5463	59,249	2 (Hangzhou, Ningbo)	2	1 (3A)	1 (3A)
Jiangsu	7899	62,290	2 (Nantong, Xuzhou)	3	1 (3B or less)	2 (3A)
Liaoning	4383	50,760	2 (Shenyang, Tieling)	2	1 (3A)	1 (3A)
Central region						
Hebei	7241	33,969	1 (Tangshan)	2	2 (3A)	0
Henan	9388	28,661	1 (Zhengzhou)	1	0	1 (3A)
Hunan	6596	29,880	1 (Changsha)	1	0	1 (3A)
Heilongjiang	3834	32,819	2 (Harbin, Daqing)	4	2 (3A)	2 (3A)
Western region						
Xinjiang	2209	30,087	1 (Urumchi)	1	0	1 (3A)
Chongqing	2919	34,500	1 (Chongqing)	1	0	1 (3A)
Gansu	2564	19,595	2 (Lanzhou, Jinchang)	8	6 (3B or less)	2 (3A/3B or less)
Overall	–	–	22	37	22	15

^a^Based on China Statistical Yearbook 2012, avaliable from http://www.stats.gov.cn/tjsj/ndsj/2012/indexch.htm;

^b^Including Guangzhou, Shenzhen, Dongguan, Foshan and Zhongshan

### Medical expenditure data and inclusion/exclusion criteria

The inpatients simultaneously fulfilling the following conditions were included: (1) diagnosed with lung cancer as the primary tumor; (2) main treatment and expenses occurred in the investigated hospitals; (3) last discharge date was between January 1, 2002, and December 31, 2011; and (4) patients’ basic information, expenditure information, and clinical information (clinical diagnosis, treatment programs, and pathological information) were available and intact.

Individuals who had two or more primary cancers were excluded. Patients who only received a diagnosis or postoperative follow-up in the investigated hospitals were also excluded.

### Data analysis

All expenditures were reported in China Yuan (CNY) based on the value in 2011 and inflated according to China’s year-specific health care consumer price index [13]. Medical expense data were log transformed. The Student’s *t*-test and an analysis of variance were performed to compare differences between groups. All data were analyzed using SAS 9.3. Values of *P* < 0.05 were considered statistically significant.

## Results

### Sample characteristics

A total of 15,437 lung cancer patients for the period 2002–2011 were included in the analysis (Table 2). The eastern, central, and western regions accounted for 59.52, 23.66, and 25.81% of the sample, respectively. Moreover, 69.83% of the patients were from specialized hospitals, and 93.16% of the patients were from three hospitals. The mean age was 59.53 years, and 69.86% were men.

**Table 2:** Characteristics of included lung cancer cases, 2002–2011

Variable	Results (n = 15,437)	
Region^a^, n (%)		
East	7799	(50.52)
Central	3653	(23.66)
West	3985	(25.81)
Hospital type, n (%)		
General	4658	(30.17)
Specialized	10,779	(69.83)
Hospital level, n (%)		
3 A	14,381	(93.16)
3 B or less	1056	(6.84)
Gender		
Male	10,786	(69.87)
Female	4651	(30.13)
Age at diagnosis, years, mean ± SD	59.53 ± 11.19	
Age at diagnosis, years		
< 45	1537	(9.96)
45–54	3373	(21.85)
55–64	5116	(33.14)
≥ 65	5411	(35.05)
Pathological type^b^		
Squamous cell carcinoma	5380	(38.61)
Adenocarcinoma	4943	(35.47)
Others	3611	(25.91)
Clinical stage^b^		
I	2922	(18.93)
II	2344	(15.18)
III	4507	(29.2)
IV	5005	(32.42)
Not reported	659	(4.27)
The proportion of morphological verification, %	12,291	(79.64)
Number of clinical visits per case, Median (P5-P95)	1 (1–8)	
Number of clinical visits per case		
1	8975	(58.14)
2	2182	(14.13)
3	1461	(9.46)
4 +	2819	(18.26)
Number of length of stay per case, Median (P25-P75)	26 (14–55)	
Type of therapy		
Surgery	2873	(19)
Radiotherapy	1159	(7.67)
Surgery & Radiotherapy	95	(0.63)
Chemotherapy	4289	(28.37)
Surgery & Chemotherapy	2627	(17.37)
Radiotherapy & Chemotherapy	1486	(9.83)
Palliative care	1923	(12.72)
Others	668	(4.42)
% of cases with any comorbidities	5098	(33.02)
% of cases with any complications	1431	(9.27)

^a^China is divided into eastern, middle and western regions according to economic development and geographical position differences within country

^b^Clinical stage is divided into four stages according to the combination of neoplastic TNM classification. Stage I is the mildest, which mean that the lesion was localized to the mucosa or submucosa without lymph node metastasis. Stage IV is the most sever, which mean that the lesion involves peripheral organs with distant lymph node metastases or distant metastases

Squamous cell carcinoma and adenocarcinoma were diagnosed in 5,380 (38.61%) and 4,943 (35.47%) patients, respectively (Table 2). Patients diagnosed at early clinical stage I accounted for 18.93% of the total. Cases diagnosed at this stage were less than those for stage III and IV (32.42%). A substantial portion (33.02%) of the patients had other concomitant diseases, and 9.27% of the patients had complications during treatment.

The median number of clinical visits per case was one (Table 2). The number of hospitalizations per patient was either 1, 2, 3, or 4 or more, accounting for 58.14, 14.13, 9.46, and 18.26, respectively. The median length of stay in a hospital was 26 days.

Regarding therapeutic schemes, patients undergoing chemotherapy accounted for 28.37%, followed by lung surgery (19.00%) and surgery combined with chemotherapy (17.37%). Palliative therapy accounted for 12.72%.

### Medical expenditure for lung cancer diagnosis and treatment

**Fig. 1:**
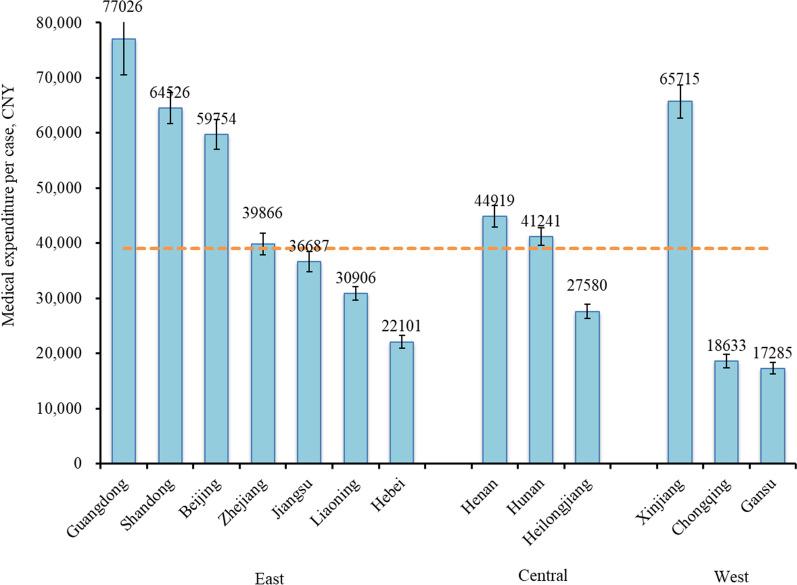
Medical expenditure for lung cancer diagnosis and treatment per case in China, by province

The average medical expenditure per patient during 2002–2011 for hospital care was 39,015 CNY (US$6,041) (Table 3). For the last three years, the average expenditure was 44,809 CNY (US$6,937) (Table 3). Expenditures for lung cancer patients were significantly associated with age at diagnosis, region, hospital type, hospital level, clinical stage, type of therapy, number of clinical visits per case, number of bed days per case, and accompanying diseases (*P* < 0.001). Patients receiving treatment in 3 A hospitals had higher expenditures (40,173CNY) (US$6,220) than those receiving treatment in the non-3A hospitals (23,246CNY) (US$3,599). The expense gaps between hospital levels increased for the period 2009–2011. The average expenditure per visit was 19,317 CNY (US$2,991). The medical expenditure for adenocarcinoma was higher than that for squamous cell carcinoma or other types. Expenditures of lung cancer patients ranged from 36,413 CNY(US$5,638) in the stage I group to 41,069 CNY (US$6,359) per capita in the stage III group, with the highest medical expenditure incurred by stage III patients (Table 4). Stage IV expenditure was the lowest among expenditures for all stages for the period 2009–2011. There were also significant regional differences (17,285CNY–77,026 CNY) (US$2,676-US$11,926), with the lowest expenditures in the western regions and the highest in eastern regions. The expenditures of all 13 provinces are shown in Fig. 1.

**Table 3:** Subgroup analysis of medical expenditure for lung cancer diagnosis and treatment per case

Variable	Expenditure per case during 2002–2011, CNY Mean (95% CI)	Value	*P*	Expenditure per case during 2009–2011, CNY Mean (95% CI)	Value	*P*
Overall	39,015(38,401–39,629)			44,809 (43,656–45,962)		
Region		378.00	< 0.001^a^		223.69	< 0.001^a^
East	43,100(42,185–44,015)			51,414 (49,721–53,107)		
Central	38,003(37,014–38,992)			46,151 (44,045–48,257)		
West	31,948(30,701–33,196)			32,439 (30,347–34,531)		
Hospital type		− 17.94	< 0.001^b^		− 4.61	< 0.001^b^
General hospital	32,043(31,054–33,031)			43,182 (41,131–45,232)		
Specialized hospital	42,028(41,266–42,790)			45,637 (44,245–47,029)		
Hospital level		19.95	< 0.001^b^		14.83	< 0.001^b^
3 A	40,173(39,531–40,815)			46,192 (45,000–47,383)		
3 B or less	23,246(21,457–25,034)			18,660 (15,710–21,611)		
Number of clinical visits per case		1671.52	< 0.001^a^		408.03	< 0.001^a^
1	23,870(23,380–24,360)			26,696 (25,698–27,693)		
2	45,966(44,430–47,501)			48,008 (45,276–50,739)		
3	59,332(57,165–61,499)			59,473 (55,859–63,087)		
4 +	71,323(69,462–73,183)			70,397 (67,671–73,122)		
Gender		6.18	< 0.001^b^		1.57	0.116^b^
Male	40,035(39,287–40,784)			45,285 (43,885–46,684)		
Female	36,649(35,582–37,715)			43,766 (41,732–45,799)		
Age at diagnosis, years		8.35	< 0.001^a^		9.41	< 0.001^a^
< 45	38,198(36,210–40,186)			43,750 (39,551–47,949)		
45–54	39,202(37,913–40,491)			47,391 (44,746–50,036)		
55–64	40,139(39,070–41,208)			46,288 (44,401–48,176)		
≥ 65	38,068(37,026–39,110)			42,152 (40,236–44,068)		
Pathological type		67.80	< 0.001^b^		32.21	< 0.001^b^
Squamous cell carcinoma	39,154(38,189–40,119)			47,491 (45,509–49,473)		
Adenocarcinoma	43,416(42,280–44,552)			50,693 (48,676–52,710)		
Others	36,854(35,548–38,161)			42,886 (40,259–45,474)		
Clinical stage		21.03	< 0.001^a^		21.81	< 0.001^a^
I	36,413(35,307–37,520)			42,960 (40,820–45,101)		
II	36,713(35,357–38,070)			43,851 (41,068–46,635)		
III	41,069(39,907–42,232)			45,861 (43,754–47,968)		
IV	39,904(38,694–41,115)			42,914 (40,703–45,125)		

*CNY* Chinese CNY

^a^ANOVA test after logarithm transition; ^b^ Two-sample Student’s *t* test after logarithm transition

**Table 4:** Subgroup analysis of medical expenditure for lung cancer diagnosis and treatment per case, Price in 2019

Variable	Expenditure per case during 2002–2011, CNY Mean (95% *CI*)	Value	*P*	Expenditure per case during 2009–2011, CNY Mean (95% *CI*)	Value	*P*
Overall	49,432(48,654–50,210)			56,773(55,312–58,233)		
Region		378.00	< 0.001^a^		223.69	< 0.001^a^
East	54,607(53,448–55,767)			65,142(62,997–67,287)		
Central				58,473(55,805–61,142)		
West	40,478(46,897–49,403)			41,100(38,450–43,751)		
Hospital type		− 17.94	< 0.001^b^		− 4.61	< 0.001^b^
General hospital	40,598(39,345–41,850)			54,712(52,113–57,309)		
Specialized hospital	53,249(52,284–54,215)			57,822(56,058–21,576)		
Hospital level		19.95	< 0.001^b^		14.83	< 0.001^b^
3A	50,899(50,085–51,713)			58,525(57,015–60,034)		
3A less	29,453(27,186–31,718)			23,642(19,905–27,381)		
Number of clinical visits per case		1671.52	< 0.001^a^		408.03	< 0.001^a^
1	30,243(29,622–30,864)			33,824(32,559–35,087)		
2	58,239(56,293–60,184)			60,826(57,365–64,286)		
3	75,174(72,428–77,919)			75,352(70,773–79,931)		
4 +	90,366(88,008–92,723)			89,193(85,739–92,646)		
Gender		6.18	< 0.001^b^		1.57	0.116^b^
Male	50,724(49,777–51,673)			57,376(55,602–58,027)		
Female	46,434(45,082–47,785)			55,452(52,874–58,027)		
Age at diagnosis, y		8.35	< 0.001^a^		9.41	< 0.001^a^
< 45	48,397(45,878–50,916)			55,431(50,111–60,751)		
45–54	49,669(48,036–51,302)			60,044(56,693–63,396)		
55–64	50,856(49,502–52,211)			58,647(56,256–61,039)		
≥ 65	48,232(46,912–49,552)			53,407(50,979–55,834)		
Pathological type		67.80	< 0.001^b^		32.21	< 0.001^b^
Squamous cell carcinoma	49,608(48,385–50,831)			60,171(57,660–62,682)		
Adenocarcinoma	55,008(53,569–56,447)			64,228(61,672–66,784)		
Others	46,694(45,039–48,350)			54,337(51,008–57,616)		
Clinical stage		21.03	< 0.001^a^		21.81	< 0.001^a^
I	46,135(44,734–47,538)			54,430(51,719–57,143)		
II	46,515(44,797–48,235)			55,559(52,033–59,087)		
III	52,034(50,562–53,508)			58,106(55,436–60,775)		
IV	50,558(49,025–52,093)			54,372(51,571–57,173)		

*CNY* Chinese CNY

^a^ ANOVA test after logarithm transition

^b^ Two-sample Student t test after logarithm transition

### Annual time trends in medical expenditure

**Fig. 2:**
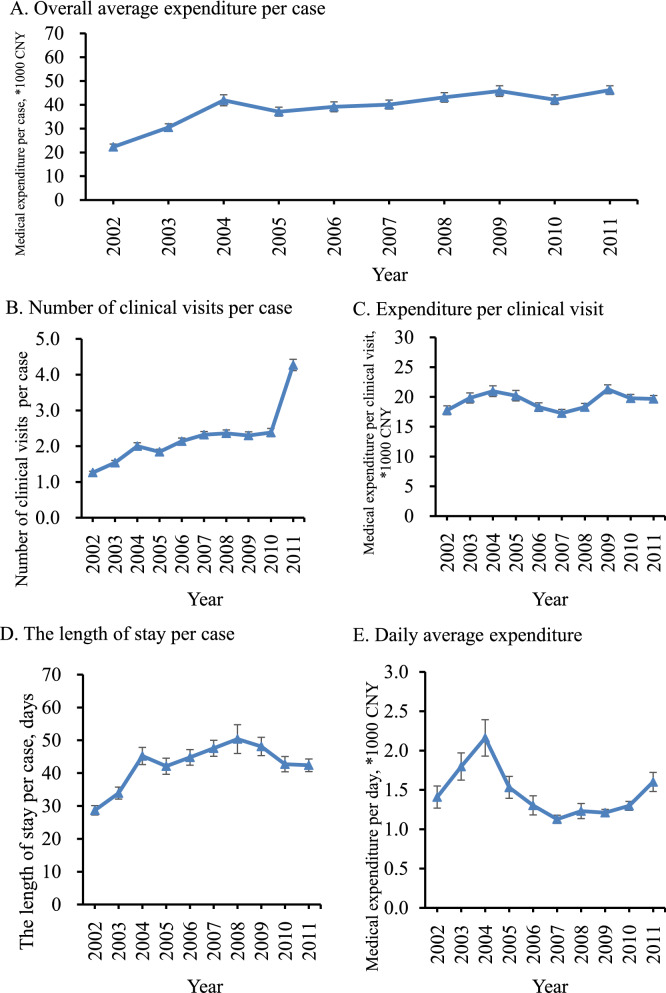
Time trend of medical expenditure and related factors for lung cancer diagnosis and treatment in China, by subgroup, 2002–2011

Overall, the average medical expenditure of lung cancer patients increased with the annual growth rate was 7.55% over the decade evaluated (Fig. 2). The average expenditure per visit had an average annual growth rate of 1.04% from 2002 to 2011, while the number of clinical visits per patient increased significantly from 1.26 in 2002 to 4.27 in 2011. The average daily medical expenditure increased at an average annual growth rate of 1.28% from 2002 to 2011. The average length of stay per case increased significantly from 28.68 days in 2002 to 42.39 days in 2011; the highest average length of stay was 50.37 days in 2008.

**Fig. 3:**
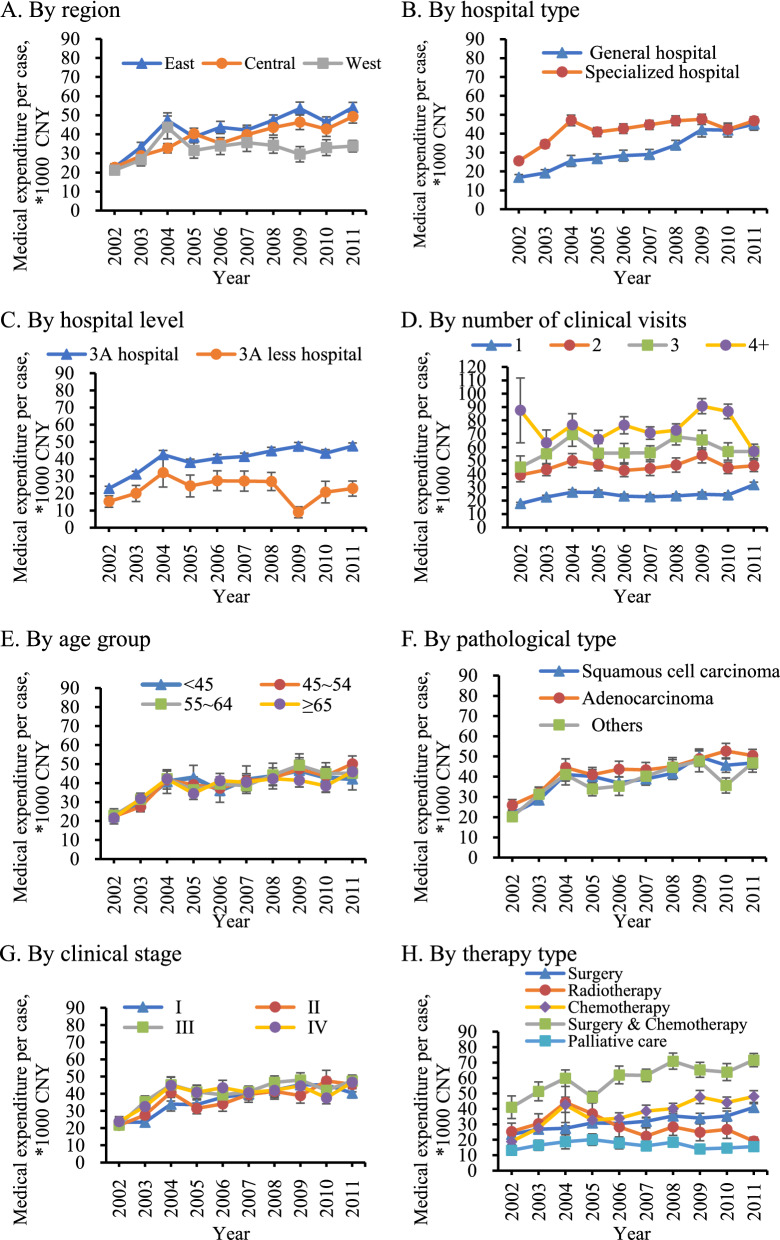
Time trend of medical expenditure for lung cancer diagnosis and treatment per case in China, by subgroup, 2002–2011

The time trends in medical expenditures per capita varied based on region, type of hospital, hospital grade, visit time, diagnostic age, and pathological type (Fig. 3). The gap between the expenditures in eastern, central, and western regions widened, with the eastern and central regions increasing rapidly. The total medical expenditure per case in the eastern region was 1.6 times that of the western region in 2011. The expense gap between general hospitals and specialized hospitals decreased gradually over time. The difference between 3 A hospitals and hospitals in other levels increased gradually. The average expenditure per case for 3 A hospitals was 2.1 times that of the other hospitals in 2011, up from 1.5 times in 2002. Chemotherapy, surgery, and surgery combined with chemotherapy expenditures grew rapidly, whereas palliative treatment expenditures increased slowly. Furthermore, radiotherapy expenditures decreased over the past decade.

### Proportions of medical expenditure by service type

**Fig. 4:**
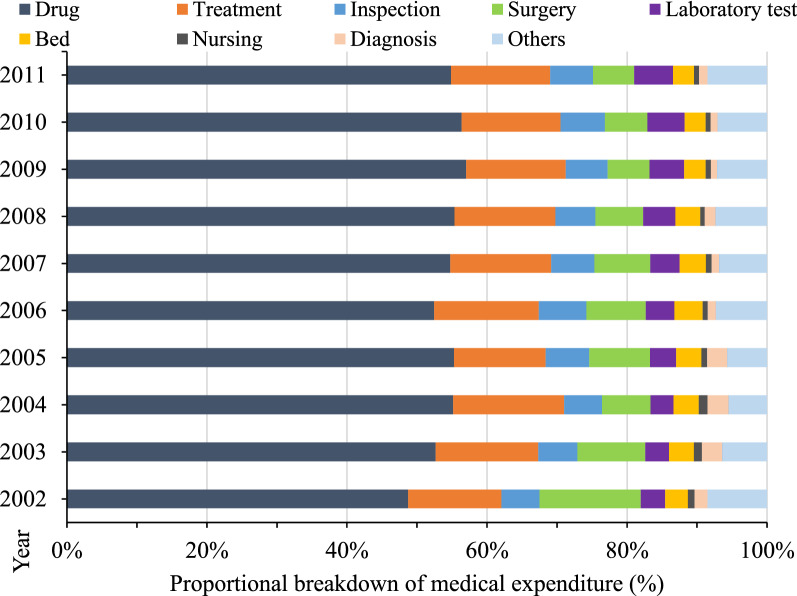
The proportional breakdown of medical expenditure for lung cancer diagnosis and treatment per case

Over half of the direct medical expenditure was for drugs, followed by treatment (treatment other than surgery and medicine), inspection, surgery, and laboratory costs (Fig. 4). Expenditure percentages for drugs, treatment, inspection, and laboratory tests increased over time (Fig. 4). The percentage of drug expenditures relative to the total medical expenditure ranged from 48.74% in 2002 to 54.85% in 2011; it peaked at 57.0% in 2009. Laboratory costs substantially increased from 3.5% in 2002 to 5.5% in 2011. Meanwhile, surgical costs relative to the total medical expenditure decreased sharply from 14.49% in 2002 to 5.88% in 2011. The share of the expenditure for beds, nursing, and inspections decreased slightly over time.

## Discussion

The average medical expenditure per patient with lung cancer was 39015 CNY (US$6,041) for the period 2002–2011. The narrow confidence interval range indicated the representativeness and robustness of the multicenter survey. This situation was consistent with other cancer studies conducted in the CanSPUC program [13, 14].It was estimated that 94% of the overall medical expenses occurred in the first year after the diagnosis of lung cancer, and was on average 36,675 CNY (US$5,678), which was higher than the per capita gross domestic product in 2011 (35,181 CNY) (US$5,447). Furthermore, it was much higher than the annual per capita disposable income of urban households in 2011 (21,810 CNY) (US$3,377) [15]. Therefore, compared to an individual’s income, medical costs of lung cancer were substantial, potentially leading to a high probability of catastrophic health payments.

A previous multi-center study on lung cancer direct medical expenditure published in 2021 measured the direct medical expenditure of enrollees using claim data of urban basic medical insurance between 2013 and 2016[16]. The consequent estimate of inpatient expenditure was 34,240 CNY (US$5,414) per lung cancer patient which was slightly lower than our survey. The difference might be due to different survey methodology applied and period covered.

Medical expenses for patients with lung cancer grew continually over time. The annual average growth rate in medical expenditure per patient was 7.55% for the period 2002–2011. 2010. This trend was consistent with the change in the length of stay in the hospital, which declined from 48.13 days in 2009 to 42.39 days in 2011, implying that reducing the average number of days of hospitalization was effective in decreasing patient costs.

Medical expenses for patients with lung cancer were influenced by many factors. Significant differences in patient costs were found among different regions and provinces. Medical expenditures in the central and eastern regions were significantly higher than those in the western region, a finding that was consistent with local economy levels. The differences might have been caused by diverse medical technology levels and medical service prices, which are fixed by provincial administrations according to the regional economy and health service costs. The regional gap of medical expenditure continued to widen over ten years.

Medical expenditures also varied by clinical stage. Expenditures of patients with stages III and IV lung cancer were higher than those of patients with stages I and II diagnoses. Early-stage patients accounted for 34%, with the majority of patients (62%) diagnosed at an advanced stage. Only 37% of patients received surgical treatment. Some patients might already have missed potential surgical cures and were only indicated for long courses of radiation and chemotherapy or palliative care, generally leading to unfavorable results and a long-term financial burden [17]. Thus it is essential to conduct early detection and treatment for lung cancer through screening to reduce the disease burden [18]. The large-scale CanSPUC program promotes early diagnosis and treatment of cancer in China.

The time trends in medical expenditures varied by treatment strategy. Expenditures of patients who underwent chemotherapy or surgery combined with chemotherapy increased rapidly, which might be attributed to the rise in prices of antineoplastic drugs. There were no significant increases in expenditures for radiation therapy and palliative treatment because radiotherapy prices remained stable, thanks to the supervision of medical services administrators.

Regarding medical expenditures of patients with lung cancer, drug expenditures accounted for the largest proportion and increased over time. This finding is consistent with other domestic research results regarding lung cancer costs [7, 19]. Laboratory and inspection expenditures have continued to rise over the past decade. In contrast, expenditures for surgery, nursing care, and diagnoses (reflecting the value of medical labor) were relatively low in terms of the overall medical expenditure. The proportion of surgical expenses relative to the total medical expenditure decreased from 14.5% in 2002 to 5.9% in 2011. Less than 1% of the total expenditure was attributed to nursing expenses, and this percentage has been falling over the past decade. The long treatment course for patients with lung cancer requires a large number of antineoplastic and supportive drugs. On the one hand, rapid advances in innovative drug and diagnosis technologies in recent years have further increased the cost of drugs [20, 21]. On the other hand, hospitals’ drugs policies and economic incentives could lead to a supplier-induced demand for physicians and an increase in the use of drugs and high-tech inspections [22]. It is anticipated that the trend of increasing medical expenditures could be diminished by new health care reform measures in China regarding financing and the drug policies of public hospitals [23, 24].

The direct medical expenditure associated with lung cancer might be an underestimation based on the following limitations. First, it was difficult to collect all medical record information of the patients in the selected hospitals, especially outpatient records. Some hospitals had not implemented electronic medical records management for previous years. Second, the diagnosis and treatment expenditures of patients with lung cancer used in this study were only from selected hospitals; expenditures of patients enrolled in other hospitals were not included. Thus, the complete direct medical cost incurred by patients with lung cancer might not be reflected in the expenditure estimate. Third, over-the-counter pharmaceuticals or prescriptions filled at pharmacies were not included in our estimates since only hospital expenses were included. A single-center study regarding the economic burden resulting from lung cancer indicated that over-the-counter pharmaceuticals accounted for 26.2% of the direct medical cost [9]. Forth, the vast majority of patients came from general hospitals or tumor specialized hospitals of 3 A level, which potentially decreased the generalization of this data. Finally, this study aimed to estimate the direct medical cost of lung cancer based on the medical records. Indirect and intangible costs were not included.

## Conclusions

The overall medical expenditure incurred for lung cancer diagnosis and treatment was substantial and rose rapidly in China. Reducing the average length of hospital stay could be beneficial for decreasing the total expenditure. Drugs, treatment, inspection, and surgery accounted for most of the total expenditure of lung cancer care. The largest component was the expenditure for drugs. Reducing drug costs is one of the means that can reduce the economic burden of lung cancer patients. Additionally, more attention should be paid to screening for lung cancer to facilitate detection at an early stage, and thus reduce relevant treatment costs.

## Data Availability

The data sets used and/or analysed during the current study are not publicly available due to the program requirement on data confidentiality.

## Abbreviations

CanSPUC: Cancer Screening Program in Urban China
CNY: China Yuan
DALYs: Disability-adjusted life-years
CI: Confidence interval
